# Pedestrian Detection in Far-Infrared Daytime Images Using a Hierarchical Codebook of SURF

**DOI:** 10.3390/s150408570

**Published:** 2015-04-13

**Authors:** Bassem Besbes, Alexandrina Rogozan, Adela-Maria Rus, Abdelaziz Bensrhair, Alberto Broggi

**Affiliations:** 1 Diotasoft, 15 Boulevard Emile Baudot, Massy 91300, France; E-Mail: bbe@diotasoft.com; 2 LITIS Laboratory, National Institute of Applied Sciences, 76801 Saint-Etienne-du-Rouvray Cedex, France; E-Mail: abdelaziz.bensrhair@insa-rouen.fr; 3 Faculty of Computer Science, Babes-Bolyai University, Kogalniceanu no.1, Cluj-Napoca RO-400084, Romania; 4 Dipartimento di Ingegneria dell' Informazione, Universita di Parma, Parco Area delle Scienze, Parma 181/a 43124, Italy; E-Mail: broggi@vislab.it

**Keywords:** pedestrian detection, far-infrared images, scale-invariant feature matching, SURF, hierarchical codebook, SVM, pedestrian classification and tracking

## Abstract

One of the main challenges in intelligent vehicles concerns pedestrian detection for driving assistance. Recent experiments have showed that state-of-the-art descriptors provide better performances on the far-infrared (FIR) spectrum than on the visible one, even in daytime conditions, for pedestrian classification. In this paper, we propose a pedestrian detector with on-board FIR camera. Our main contribution is the exploitation of the specific characteristics of FIR images to design a fast, scale-invariant and robust pedestrian detector. Our system consists of three modules, each based on speeded-up robust feature (SURF) matching. The first module allows generating regions-of-interest (ROI), since in FIR images of the pedestrian shapes may vary in large scales, but heads appear usually as light regions. ROI are detected with a high recall rate with the hierarchical codebook of SURF features located in head regions. The second module consists of pedestrian full-body classification by using SVM. This module allows one to enhance the precision with low computational cost. In the third module, we combine the mean shift algorithm with inter-frame scale-invariant SURF feature tracking to enhance the robustness of our system. The experimental evaluation shows that our system outperforms, in the FIR domain, the state-of-the-art Haar-like Adaboost-cascade, histogram of oriented gradients (HOG)/linear SVM (linSVM) and MultiFtrpedestrian detectors, trained on the FIR images.

## 1. Introduction

The recent advances of intelligent transportation systems and the increasing requirements for road safety have resulted in progressive integration of advanced driver assistance systems (ADAS). Over the past few years, various ADAS have been implemented into cars, including adaptive cruise control, lane departure warning, blind spot detection, among other intelligent functions. These systems allow one to increase road safety by helping the driver in his or her driving process. For intelligent vehicles, the implementation of pedestrian protection systems (PPS) is necessary to reduce the number of accidents and fatalities. Night vision systems are currently available in high-end cars, such as the BMW 7-series. These on-board systems have to be real time, precise and robust during day and nighttime in order to detect pedestrians, assist the driver and even intervene for avoiding accidents.

PPS require a robust pedestrian detection system in order to cope with variable pedestrian shapes and appearances, difficult lighting conditions and cluttered backgrounds. In the last few years, many research systems have been proposed to tackle these problems. The existing systems process data acquired by passive sensors, either in monocular [1,2] or stereo [3,4] configurations recorded with visible or infrared cameras, active sensors [5,6] and even with sophisticated and expensive system configurations combining active sensors with passive cameras [7,8]. Within such systems, passive vision seems to us a natural choice, because it mimics human perception and allows the recognition of the obstacle types (pedestrian, cycle, vehicle).

The basic architecture of pedestrian detection systems is generally composed of three modules: ROI generation, hypothesis validation and pedestrian tracking. Recent reviews are given in [9–12]. For on-board monocular vision systems, the problems of ROI generation and pedestrian tracking are particularly difficult, because road scenes are highly dynamic. As the pedestrians and the camera are moving, techniques, like simple foreground or motion modeling [13], could not be reliably applied. Moreover, information about disparity or 3D pixel position is not always available to simplify the process of ROI generation [3]. The simplest technique to obtain initial pedestrian locations is to select all of the possible candidates in the image according to the aspect ratio and size [14] without explicit segmentation. This brings us to the inherent problem of sliding-windows for generic object detection and localization, which is usually solved with machine learning and classification techniques [1,15].

Sliding-window techniques have been widely used and experimented in public pedestrian datasets, such as Caltech and Daimler, taken with a visible (VIS) camera from a vehicle driving through an urban environment [10–12]. An interesting benchmark [11] of the most promising pedestrian detection systems on the visible spectrum was performed on the Caltech pedestrian dataset. The MultiFtr system, based on a combination of histogram of oriented gradients (HOG) features and Haar wavelets within an AdaBoost classifier [16], with a HOG-based detector with a linear SVM classifier [1] as a close second, outperforms all of the other pedestrian detection systems. A recent survey [12], having more algorithms in comparison to the Caltech database, highlights that the MultiFtr-based detectors are among the most accurate ones, although they happen also to be quite slower. In fact, MultiFtr + motion detector was the best, followed by ChannelFtr, where Haar-like features are computed over multiple channels of visual data, including LUV color channels. Another survey [10] has involved the evaluation of different systems on the Daimler pedestrian dataset. The results indicate an advantage of the system based on HOG-features with linear SVM [1] at higher image resolution and lower proceeding speed, but a superiority of Haar-like wavelets with the AdaBoost cascade approach [17] at lower image resolution and near-real-time processing. The following systems: Haar-like Adaboost cascade, HOG/linear SVM (linSVM) and MultiFtr, trained on FIR images, will therefore serve as references for evaluating our pedestrian detection system in the FIR domain.

Thanks to the decreasing cost of infrared (IR) cameras, different pedestrian detection systems processing IR images have been proposed over the past few years [18–21]. The advantage of employing this technology is that even in very low light conditions, pedestrians appear mostly as light regions in contrast to the darker background. Besides, recent experiments have shown that all benchmarked descriptors, like HOG, LBP, LGP and ISS, perform better for pedestrian classification on the FIR spectrum than on the visible one, even in daytime conditions [22]. In fact, images acquired with IR cameras could be less impacted by illumination, shadow, color and texture, compared to visible ones. In the literature, one distinguishes between near-IR (NIR, 0.75–1.4 µm) and far-IR (FIR, 7–14 µm) pedestrian detection systems. Since NIR images are quite similar to daylight VIS ones, they seem more appropriate than FIR for ADAS applications, like lane departure warning and traffic sign recognition [9]. As we focus our interest on pedestrian detection applications, we decided to use an FIR camera, which is not only the appropriate choice during the night or in low-light conditions, but is also very convenient for distinguishing warm targets, like pedestrians, in daytime conditions. However, FIR images have characteristics that make the detection still a difficult task. Firstly, pedestrian image intensities are not uniform, since they vary with respect to temperature, distance, orientation and object surface properties. Secondly, FIR images can be also characterized by blur and a lack of texture information. Thirdly, objects, such as animals, electric boxes and light poles, produce additional contrast, which complicates the pedestrian detection process. Moreover, FIR images are hard to exploit in summer, where the temperature between pedestrians and other objects is very low. Our goal is therefore to overcome these difficulties by exploiting the specific characteristics of FIR images in order to design a fast, scale-invariant and robust pedestrian detection system in daytime conditions.

The outline of the paper is as follows. After reviewing related work in Section 2 and emphasizing our contributions in Section 3, we introduce the background of our detection system in Section 4. Our pedestrian representation method, based on the conception of an original hierarchical codebook, is described in Section 5. Section 6 covers the details of our pedestrian detection system. Experimental results that validate our system are presented in Section 7. Finally, conclusions are drawn, and future work is suggested.

## 2. Related Work

Since pedestrians are represented by higher intensity than the background in FIR images, it is feasible to generate ROI by thresholding the images appropriately [18,20]. Bertozzi *et al.* [21] proposed an adaptive threshold by assuming that non-pedestrian intensities follow a Gaussian distribution. In [19], thresholding was performed using histogram analysis. Nevertheless, the problem of choosing an appropriate threshold is a key issue, not only for the additional light produced by non-pedestrian objects, but also because pedestrian intensities vary with respect to temperature and distance. In [23,24], the pedestrian localization process consists of searching for warm symmetrical objects with a specific size and aspect ratio. Mahlisch *et al.* [25] proposed a shape-based multiple detector for low-resolution FIR pedestrian recognition. To speed up processing, image regions that pass a relaxed “flat-world-assumption” are applied to both a hierarchical contour matching and a cascade classification, according to the likelihood-image estimated by a hyper-permutation network. Given the typical smooth edges in FIR data and the difficulty of edge segmentation, the shape-dependent methods seem to us not suited for precise localization of pedestrians in FIR images. Moreover, shape-dependent methods could not overcome the problem of occlusions.

Meis *et al.* [26] addressed the occlusion problem by stating that pedestrian groups and, in particular, pedestrians walking side by side can be separated by using a head detector. In order to cope with large intra-class variation, Jungling and Arens [2] proposed to learn an implicit shape model (ISM) of pedestrians in IR images. The basic theory of this approach was presented in [27,28]. It consists of defining allowed shapes implicitly where local appearances, represented in a codebook structure, are consistent with each other. The advantages of this approach are its flexibility to face the problem of pedestrian variability and the fact that a small number of training examples is needed to learn an efficient shape model.

In ROI generation, algorithms robust to partial occlusions and large intra-class variations tend to produce a significant number of false positives. Techniques, like symmetry verification [29] or template matching [30], are not precise enough to avoid false positives, nor to recognize pedestrians in particular situations (walking, running, occluded). These problems have been especially addressed by integrating pattern representation and classification methods to reliably recognize pedestrians [14,18,20,31].

Most pedestrian classification approaches are based on global or region object representation. The global features are computed over all of the pixels within a bounding box (BB), while region-based features are determined over image regions. For instance, gray-level image features [18] and Gabor wavelets [31,32] are based on global representations. Typical region-based techniques include Haar wavelets [14,33] and histograms of oriented gradients (HOGs) [20]. These representations involve the influence of every pixel that lies within a BB, which may cause difficulties for accurate object classification. In fact, a BB does not capture explicitly the true shape of the object inside it and could even contain a majority of non-object pixels. This is especially problematic for pedestrians, which are heavily occluded by other road objects. Besides, it is almost impossible to define an explicit global model for all possible shapes and appearances of pedestrians. Solutions based on local descriptors [34,35], extracted around a set of interest points for matching and recognition, could resolve these problems.

Since SVM has shown impressive learning and classification performance, there has been an increasing interest in developing algorithms that combine image representation by local descriptors with SVM [36–38]. While global- and region-based features extracted from images resized at the same resolution generate a fixed dimension feature vector, local features cannot be extracted with the same number and at the same location in images. Thus, the main problem to solve is to define an SVM kernel able to compare objects with different number of mismatch features. In the literature, many techniques have been proposed to overcome this problem. Different methods based on histograms built on local feature clusters were proposed. The histogram counts, for each object, how many feature points of each codebook cluster index occur [38–40]. Wallraven *et al.* [36] introduced a formulation of feature matching step as a part of the kernel itself, known as local matching kernel (LMK). Inspired by this technique, Fritz *et al.* [37] proposed a suitable combination between codebook representation with SVM classification and added stronger spatial constraints by imposing position rules for feature matching. LMK has good classification performance, but presents high computational cost to compute feature matching. In [41], we showed that a significant speed up in classification time can be achieved without losing performances by using traditional kernels, like linear (linSVM) or RBF (RBF-SVM) on a SURF-based, compact, but discriminative signature of pedestrians.

The ROI generation and classification modules are usually configured to detect pedestrians from a given image frame. Several pedestrian detectors involve a tracking module, which is usually based on hard-costing Kalman or particular filters to follow detected pedestrians over time [9,12]. This step has several purposes: avoiding false detections over time, predicting future pedestrian positions, thus feeding the foreground segmentation algorithm with pre-candidates, and, at a higher level, making useful inferences about pedestrian behavior (e.g., walking direction). However, the detected hypotheses could be integrated in a fast tracking module, which keeps track of pedestrians in successive frames.

## 3. Contributions

With respect to the related works, our contributions stem from how we:

- design an algorithm for automatic generation of a pertinent head codebook and flexible enough to tolerate important pedestrian appearance changes;
- define a common hierarchical representation that allows not only to detect pedestrian head, but also pedestrian body recognition and tracking;
- exploit the hierarchical structure of the codebook to further improve time processing, detection and classification performance;
- propose a temporal SURF feature matching for pedestrian tracking in successive frames.

We introduce in the next section the background of our system before describing its basic components.

## 4. Background of the System

In this section, we describe the techniques of image representation by SURF features and SVM-based object classification.

### 4.1. Image Representation by SURF Features

As we previously mentioned, image representations by local features have been shown to be robust to occlusions and background changes. Among these, we choose to use SURF [35], because it is a scale- and rotation-invariant point of interest (POI) detector and descriptor. Bay *et al.* [35] demonstrated that the SURF detector is several times faster than SIFT [34] and more robust against different image transformations. The SURF descriptor is based on sums of 2D Haar wavelet responses and makes an efficient use of integral images. The SURF characteristics that we exploit are:

- Haar wavelet descriptor of 64 elements,
- Scale of the descriptor (ρ),
- Hessian value (*H*) used to approximate the feature strengths,
- Laplacian value, which is negative for light regions and positive for dark ones.

This last characteristic is especially useful for pedestrian detection. In fact, a pedestrian head mostly appears in an FIR image as a light region in contrast to the darker background. Thus, in the learning phase, we retained only SURF points with a negative Laplacian value to build our head codebook representation. In the testing stage, codebook matching with POI extracted from an image allows one to generate pedestrian hypotheses. Then, these hypotheses are validated by using an SVM classifier. This supervised classification technique is introduced in the next section.

### 4.2. SVM-Based Object Classification

In recent years, there has been considerable interest in combining image representation using a codebook with SVM. We distinguish between two approaches: codebook combination with kernels based on histograms [38–40,42] and respectively specialized local matching kernels, called LMK, suitable for local feature matching [37]. Basically, histograms computed from the codebook matching count how many feature points of each codebook cluster index occur. Then, the scalar product (linSVM), Gaussian distance (RBF-SVM), *χ*^2^ similarity measure (*χ*^2^-RBF) [39] or histogram intersection (HI-SVM) [40] between two histogram bins could be used within an SVM kernel. The definition of these kernels is given in Table 1, where *h_i_* and $h_{i}^{\prime }$ are the values taken on by the histogram-vector *X* and *Y* at the bin numbered *i*.

A recipe for constructing kernels, suitable for object recognition with local features, was proposed in [36]. These kernels were called local matching kernels (LMK), because the feature matching step is seen as a part of the kernel itself. The LMK kernels are performant, but present high computational cost to compute feature matching. Let *X* and *Y* be two sets of local features with $X={\left\{\vec{x_{i}}\right\}}_{i=1}^{n1}$ and $Y={\left\{\vec{y_{i}}\right\}}_{j=1}^{n2}$. Let $A=\{\vec{A_{1}},\ldots ,\vec{A_{n_{1}}}\}$ be their corresponding codebook activations in the set of *N* clusters where $\vec{A_{i}}=\{a_{1},\ldots ,a_{N}\}$. Thus, the complexity is ( 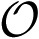)(*n*1 × *n*2 × *N*^2^). In fact, the LMK approach evaluates all combinatorial possibilities to match local features from two objects. Therefore, rather than an LMK approach, we prefer a codebook combination with kernels based on histograms. Our proposal consists of using an original hierarchical codebook (HC)-SURF representation to feed a RBF-SVM kernel. Experiments show that our method, when compared with all previous state-of-the-art kernels, including LMK, allows for improvement of the classification performance.

After introducing the background of our system, we develop our proposal in the following sections.

## 5. Hierarchical Codebook of SURF

In our system, the variability of pedestrians is represented by learning, in the first step, a codebook of local head appearances.

### 5.1. Hierarchical Codebook Generation

Clustering is a common method for learning a compact and discriminative codebook. We choose to use reciprocal nearest neighbor (RNN) search [28] for SURF descriptor clustering in order to reduce both time and space complexity [28,43]. Starting with each SURF descriptor seen as an unitary cluster, agglomerative clustering is performed: the two most similar clusters are merged together if the Euclidean distance between their descriptors is below a threshold. In order to solve the problem of cluster thresholding, we propose an hierarchical structure where each level presents a specific clustering threshold. This tree structure allows one to tolerate different degrees of local appearance variability. In order to estimate the optimal tree structure and the level-specific thresholds, we propose an evaluation function. Following, all SURF features extracted from pedestrian heads are represented in a tree structure. Each tree level contains clusters, represented like nodes, resulted from a clustering with a specific level threshold value. Each node *i* is characterized, besides its level on the tree, by:

- the centroid *C_i_*, which is the mean of all SURF vector descriptors,
- the radius *R_i_*, which represents the Euclidean distance from the center to the furthest feature in the cluster *i*,
- the mean *r̄_i_* of the *r_ik_* values corresponding to the descriptor *k* contained in the cluster *i*,
- the number of interest points *n_i_* contained in the cluster.

The radius of clusters in the bottom levels is below the initial clustering threshold *t*_1_, while the top node radius is below *t_max_*. This *t_max_* value is fixed as the smallest value that allows one to group all SURF descriptors together into a single cluster. We emphasize that the optimal values of the initial clustering threshold *t*_1_ and then the number of tree levels (*l*^θ^) could be both obtained by minimizing a unique clustering evaluation function *F*(*t*,*l*). Through a literature review, we found that most of the evaluation methods, related to clustering purposes, introduced a criterion related to multi-class tasks for heterogeneous data. A brief discussion of these methods, defining several evaluation criteria, is given in [44]. Our HC concerns homogeneous data, since it collects only head pedestrian descriptors. For that, a typical evaluation function leads to minimizing the intra-cluster distance, as well as maximizing the distance between clusters. We consider that this criterion is already inherent to the RNN algorithm [28]. We propose to consider for our evaluation function another criteria related to the hierarchical structure. This function *F*(*t*, *l*), given in Equation (1), allows one to minimize the number of unitary clusters by presenting different clustering thresholds. Note that unitary clusters are those never merged at any level of the tree, and thus, still remain only at the bottom level. Moreover, the function *F* allows also to ensure that the resulting clusters are compact and, hence, to determine the optimal hierarchical structure. Finally, this leads to automatic codebook generation via solving the problem of cluster thresholding.

Let *N*^1^ be the total number of bottom codebook clusters located in the first level (*l* = 1). *N*^1^ includes $N_{u}^{1}$ unitary clusters (*n_i_* = 1) and $N_{nu}^{1}$ non-unitary ones (*n_i_* > 1).

(1) $$F(t,l)=(Q_{1})\times (Q_{2})=(\frac{N_{u}^{1}}{N^{1}})\times (\frac{\max_{i\in 1..N^{1}}(n_{i})}{\sum_{i=1}^{N^{1}}n_{i}})$$

$$\begin{array}{ll} t_{1} & =\arg\underset{t\in \mathbb{R}}{\min}F(t,1)\\ l^{\theta } & =\arg\underset{t\in \mathbb{N}}{\min}F(t,1) \end{array}$$

where *t*_1_ is the optimal value of the initial clustering threshold and (*l*^θ^) the optimal number of tree levels.

Minimizing *F* is equivalent to minimizing *Q*_1_ and *Q*_2_. *Q*_1_ tends to minimize the number of unitary clusters. *Q*_2_ is used to reinforce the balance between cluster sizes.

Once the optimal value *t*_1_ is estimated, the HC depth is increased iteratively until reaching a local minimum F(*t*_1_, *l*^θ^). When incrementing tree levels, we choose to increase the threshold from bottom to top by doubling the pitch between two consecutive levels. Once the tree is built, clusters that were never merged in any level of the tree are removed from the codebook. This allows one to compact as much as possible the pedestrian head signature without losing pertinent information.

### 5.2. Hierarchical Codebook Matching

**Figure 1:**
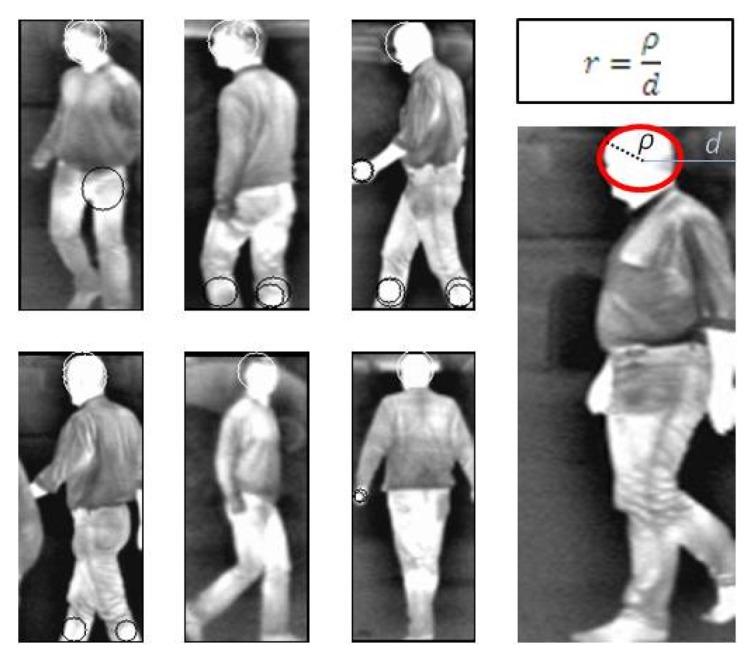
SURF extraction only from head regions (white circles).

Given a test image, we extract the SURF descriptors from light regions (having negative Laplacian values), as shown in Figure 1, and match them to the HC. The process is accelerated by exploiting the specificity of our hierarchical representation. In fact, the matching is performed by first computing the Euclidean distance to the top node centroid. If the distance is smaller than the node radius, the match is stored, and then, only the children of this node are explored. The search is continued down to the bottom nodes with a recursive method.

For each extracted SURF point *k*, we propose to assign an activation value *A_i,k_* to each matched cluster with index *i*. Let *f_k_* be a particular SURF descriptor point; we propose to calculate the corresponding activation of matched clusters according to this formula:

(2) $$A_{i,k}=\exp(-{\left(\frac{c\times d(f_{k},C_{i})}{R_{i}}\right)}^{2})$$

where *d* is the Euclidean distance in descriptor space and *c* is an adjustment coefficient designed to increase the convergence speed of *A_i,k_* to zero. *A_i,k_* is a decreasing function with respect to *d*(*f_k_*, *C_i_*). This formulation is perfectly consistent with the principle of partial exploration of the tree, since we assign zero activation values to unexplored nodes. This highlights the interest of the hierarchical structure that has the potential to significantly speed up the codebook matching time.

It is important to note that the activation values were normalized by level. Let *N^l^* be the number of clusters located at the same hierarchical level (*l*) as the cluster *i*; the activation value *A_i,k_* is then normalized by $\sum_{j=1}^{N^{l}}A_{j,k}$:

(3) $$a_{i,k}=\frac{A_{i,k}}{\sum_{j=1}^{N^{l}}A_{j,k}}$$

In the next section, we will emphasize how the codebook activation values will be used as a common tool for pedestrian head detecting and pedestrian body classification.

## 6. Pedestrian Detection System

**Figure 2:**
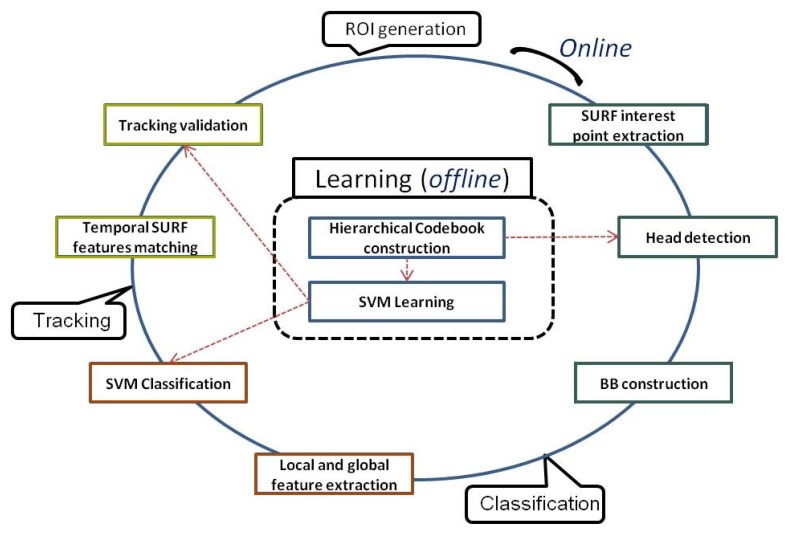
The architecture of our pedestrian detector.

This section covers the details of our pedestrian detection system. Its architecture, illustrated in Figure 2, is composed of the following three modules: ROI generation, hypothesis classification and pedestrian tracking. The dotted lines highlight the importance of the learning phase involving both HC construction and SVM training, while the red arrows indicate when they are used.

Within the ROI generation module, the SURF points are first detected all over the image, then matched to the trained clusters of HC, and then, only the matching features with heads are used to generate the candidate full-body BBs. In the classification module, designed for the validation of the candidate BBs, local and global SURF-based features are classified by SVM. Finally, in the tracking module, two steps are involved: temporal SURF feature matching and tracking validation for ROI generation improvement.

We decided to proceed with a head detecting step by using a codebook of head local appearances, but unlike the method presented in [26], based on pixel classification, our solution is based on SURF local features for matching and recognition. The SURF descriptor is particularly well suited for FIR data, where pedestrian appearance variation is rather limited compared to both the NIR and VIS spectrum. Moreover, it allows one to index and then to further consider only bright regions. The other relevant characteristic of FIR images that we have exploited is that the pedestrian head is the only area that appears usually as a light region whatever its scale and location in the image. This highlights the importance of considering only the head local appearances in codebook building, unlike the ISM technique [2]. Hence, the core part of our pedestrian detector is a highly flexible learned representation for pedestrian head appearances. This representation combines the information observed in different training examples by using a hierarchical codebook (HC). This hierarchical structure is inspired by [45], but does not require a probabilistic framework. In fact, the majority of object detection approaches based on local features formulate a voting system or detected parts assembled in a probabilistic framework [2,28,45,46]. The incorporation of a such framework requires a particularly high computationally cost to maintain belief estimates over the complete hypothesis set.

In our classification module, we investigate both local and global features based on SURF representations combined with RBF-SVM in order to recognize pedestrians in FIR images. The local features are extracted by reusing the results of codebook matching obtained in the stage of ROI generation. In other words, both the ROI generation and the classification of pedestrian hypotheses operate on a common codebook representation. Global features are also employed to provide complementary information to local appearance descriptors by characterizing shapes and textures. Finally, we propose an original tracking module, built on the mean shift algorithm [47], based on fast temporal scale-invariant feature matching.

**Figure 3:**
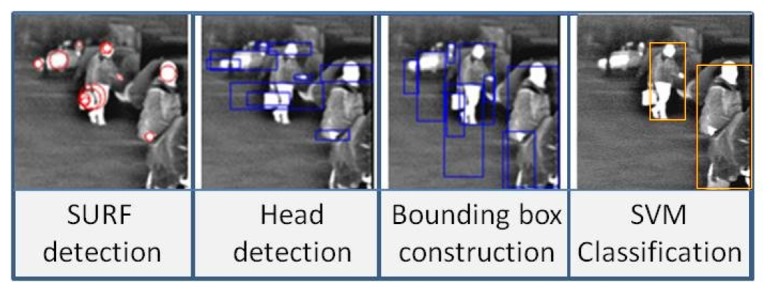
Example of pedestrian detection obtained after the ROI generation and the classification modules.

Figure 3 shows an example of pedestrian detection obtained after the ROI generation and the classification modules.

### 6.1. ROI Generation with the Hierarchical Codebook

The first component of our system is ROI generation. The main idea is to exploit the specific characteristics of FIR images where a pedestrian may vary in a large scale, but his head appears usually as a light region. Thus, we retained only SURF points with a negative Laplacian value to build our head codebook representation. The codebook is generated by collecting and then clustering, from head regions, SURF features in the learning stage. The obtained group clusters similar SURF descriptors by their Euclidean distance. In the testing stage, codebook matching with POI extracted from an image allows one to generate pedestrian hypotheses. Accordingly, we propose a robust two-step method, based on head HC code representation, where head detection is followed by full-body BB construction.

Codebook generation lies in collecting and then clustering SURF features extracted from pedestrian head regions from the training set. For each retained interest point (having a negative Laplacian value), we propose to record the ratio (*r*) between the descriptor size (ρ) and the distance *d* to the horizontal distance to the nearest BB L/Rside, as described in Figure 1. This parameter will be further used in the detection stage to suggest BB around interest points whose descriptors are similar to the centroid of the corresponding cluster.

The most important stage of ROI generation is head-codebook matching with all SURF points extracted from light regions. For each SURF point *k*, we select the codebook cluster *clus* that produces the highest activation value:

(4) $$\text{clus}=\arg\underset{i\in [1,N]}{\max}a_{i,k}$$

where the *a_i,k_* formula is given in Equation (3).

Then, an ROI of a pedestrian head, having the score of *a_clus,k_*, is generated by using the parameter *r̄_clus_* and the scale ρ*_k_* of the SURF point *k*. Since *r̄_clus_* represents the mean ratio between the scale and the horizontal distance to the nearest BB L/R side, we set the width of the head ROI to $2\times \frac{{\bar{r}}_{\text{clus}}}{\rho_{k}}$, while keeping empirically the height to the half-width value.

Since pedestrian head regions may include several POI, overlapping ROI are merged together by using a fast agglomerative clustering. To perform that, we adapt the algorithm of RNN search, used for codebook generation [28,41], to group overlapping ROI rather than similar descriptors (see Algorithm 1). This clustering process allows us to achieve a good tradeoff between precision and recall with low computational cost. The idea behind this process is to consider that the nearest neighbor of a head ROI is the one that maximizes the percentage of overlap computed by using the intersection-over-union criterion. Since the maximum percentage of occlusion considered is 50%, the clustering threshold of overlap (*t_overlap_*) is fixed to 0.5. Below this threshold, an occlusion is likely to occur; above it, a new ROI is defined, taking the weighted fusion on the pixels of the overlapping ROI, according to their scores.



**Algorithm 1** The reciprocal nearest neighbor (RNN) algorithm for overlapping ROI merging with maximum overlapping area chains.
- Start the chain *C*_1_ with a random ROI *ri* ϵ *R*{All remaining ROI are kept in *C*_2_}*last* ← 0; *lastOverlap*[0] ← 0*C*_1_ [*last*] ← *ri* ∈ *R*; *C*_2_ ← *R*\*ri***while**
*C*_2_ ≠ ∅ **do** {Search for the ROI that maximizes the overlap area}  (*o*, *overlap*) ← *getMaxOverlappingArea*(*C*_1_[*last*], *C*_2_) **if**
*overlap* > *lastOverlap*[*last*] **then**  *last* ← *last* + 1  *C*_1_[*last*] ← *o*; *C*_2_ ← *C*_2_\{*o*}  *lastOverlap*[*last*] ← *overlap* **else**  **if**
*lastOverlap*[*last*] > *t_overlap_*
**then**   *o* ← *merging*(*C*_1_[*last*], *C*_1_[*last* − 1])   *C*_2_ ← *C*_2_ ∪ {*o*}   *last* ← *last* − 2  **else**   {discard the current chain}   *last* ← −1  **end if** **end if** **if**
*last* < 0 **then**  {initialize a new chain with another ROI *ri* ∈ *C*_2_}  *last* ← *last* + 1  *C*_1_[*last*] ← *ri* ϵ *C*_2_; *C*_2_ ← *C*_2_\{*ri*} **end if****end while**


After ROI generation around head regions, pedestrian full-body BB is first roughly estimated based on the *width*/*height*ratio average, determined on the training images. Then, a new examination starts to refine the bottom edges of the BB containing the potential pedestrian by searching around the bottom border, the line which contains most of the horizontal edge points. After the construction of the whole BB, a score is given for each one based on SVM classification. Following, we remove the BB labeled as “non-pedestrians” by the classifier and resolve ambiguities from remaining overlapping ones by keeping only the BB having the highest SVM score. Our SVM classification module is described in the following section.

### 6.2. Combining Local and Global Features for SVM Pedestrian Classification

The local features are extracted using the HC and represent the local appearances of pedestrians. We have also considered several global features, since they provide complementary information by characterizing shapes and textures. Because of the large number of features, we used several ranking algorithms to select the most relevant ones [48]. The results have allowed us to select from the codebook the most effective clusters, corresponding to descriptors extracted from head regions. This finding supports our supposition of building an HC of pedestrian head appearances and its exploitation for both ROI generation and hypothesis classification. Moreover, the feature selection step allowed us to select the most relevant global features and to emphasize the complementarity between local and global ones. Finally, the combined representations are used to feed an SVM classifier.

#### 6.2.1. HC-Based Local Features

We propose to extract local features by accumulating the activation values of the HC clusters. Let *B* = {*B*_1_,…,*B_M_*} be the codebook that encompasses pedestrian appearance clusters. Let *S* = {*k*_1_,…,*k_nb_*} be a set of *nb* SURF points extracted within a given BB. We define a local feature vector *X_l_* with a function *f_local_* : *S* × *B* → ℝ that takes a set *S* of SURF points and a set of *B* clusters and returns a real value:

(5) $$f_{\text{local}}(S,B_{i})=\sum_{k=1}^{nb}a_{i,k}$$

This formulation is equivalent to a histogram voting method where each bin accumulates the activation values of a specific cluster. Finally, the local feature vector that acts as a part of the input sample of SVM is:

$$X_{l}=(f_{\text{local}}(S,B_{1}),f_{\text{local}}(S,B_{2})),\ldots ,f_{\text{local}}(S,B_{M})$$

#### 6.2.2. SURF-Based Global Features

Features extracted from the codebook characterize only the local appearance of pedestrians. In order to improve the discriminative power of pedestrian representation for classification purposes, we choose to add several fast-computing global features that provide complementary information by characterizing the shape and the texture of objects. Global feature extraction concerns only SURF points whose descriptors activate at least one cluster in the codebook.

We define a global feature vector *X_g_* with a function *f_global_* : *S* → ℝ that takes a set of SURF points *S* as an argument and returns a real value. Let us split the set *S* of SURF points, in a subset *S_LR_* of points extracted from light regions and, respectively, in a subset *S_DR_* of points extracted from dark ones. From these two subsets, we determine the following features:

- Global shape features:
  - The ratio: $\frac{\left|S_{LR}\right|}{\left|S_{DR}\right|}$ of light points over the dark ones.
  - The standard deviations of SURF descriptor sizes from *S_LR_* and *S_DR_*. We consider that looking at the scale of the SURF filter responses allows us to characterize patterns inside an object.
  - The distance from the center of gravity of *S_LR_* and, respectively, of *S_DR_* to the center of the BB. These two features provide symmetry information computed from interest point locations. They were used to characterize the shape of an object by describing the spatial layout of local descriptors.
- Global texture features:
  - The standard deviation, kurtosis and skewness of pixel intensities located inside *S* descriptors.
  - The mean and standard deviation of Hessian values of *S_LR_*.

#### 6.2.3. Pedestrian Hypotheses Validation Using SVM

The extracted local and global features are concatenated and then normalized to form a unique feature vector for the SVM classifier, which has to discriminate between pedestrian (Ped) and non-pedestrians (N-ped). Since all features are invariant to both the scale and the number of local features extracted from a given BB, our system performs the classification task without resizing images, which significantly reduces the processing time.

It is important to mention that the classification module attempts to reject false positive hypotheses from both ROI generation and tracking prediction stages. In the next section, we will show how tracking-based hypotheses are generated to further improve the robustness of our detection system.

### 6.3. Temporal Feature Matching for Tracking

This section presents the tracking module proposed to keep track of pedestrians in successive frames. We propose a simple and fast algorithm based on temporal SURF feature matching. This algorithm has two major steps: feature matching and hypotheses handling. The tracking algorithm is initialized for each detected pedestrian in image *t*. The SURF POI within a given BB are considered as the tracking features if their descriptors activate at least one HC cluster. Let *F* be the set of the tracking features of a detected pedestrian in a given image *t*. The temporal matching process, as shown in Algorithm 2, uses the method described in [34] based on nearest neighbor with ambiguity rejection method. The idea is to validate a new match only if the ratio of distances corresponding to the first minima over the distance of the second minima is lower than a threshold *t_m_*, determined by experiments.



**Algorithm 2** Feature matching using the nearest neighbor with the ambiguity rejection method.
**for** each descriptor *f_k_* ∈ *F* of an interest point (*x*, *y*) **do** 
-Define an ROI *ri* in the image *t* + 1{Search for the SURF POI that minimizes the Euclidean distance between descriptor vectors}-Search an unused descriptor *f′_j_* ∈ *F*( 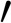) localized in *ri* giving the minimum distance *d*_1_-Search an unused POI localized in *ri* giving the second minimum distance *d*_2_ **if**
$\frac{d_{1}}{d_{2}}<t_{m}$
**then**   
-Calculate the relative position (*Pos*) of (*x*, *y*) within the bounding box outlined in the image *t*-Generate a pedestrian hypothesis in the image *t* + 1 {The hypothesis position and scale values are calculated according to *Pos* and the difference in scale between ρ*_k_* and ${\rho^{\prime }}_{k}$}  Tag *f_k_* and 
$f_{j}^{\prime }$ as used **end if****end for**


The feature matching component attempts to generate as many pedestrian hypotheses as the number of matched features. For each detected pedestrian in image *t*, the hypotheses generated in image *t* + 1 are handled in a mean shift framework. Considering prediction hypotheses at *t* + 1 as 3D votes (2D position and 1D for the scale), a score is given for each vote depending on the distance between SURF descriptor vectors. Finally, the mean shift algorithm is embedded into the 3D voting space [28] for reliable prediction of pedestrian localization. Experimental results that validate our pedestrian system are presented in the next section.

## 7. Experimental Evaluation

In this section, we present the experimental results of our system. The Tetravision image database used for our experiments is provided by the Artificial Vision and Intelligent Systems Laboratory (VisLab) of Parma University [3]. All of the images are taken with on-board stereo-vision cameras in the visible and FIR spectrum and represent daytime road scenes.

**Figure 4:**
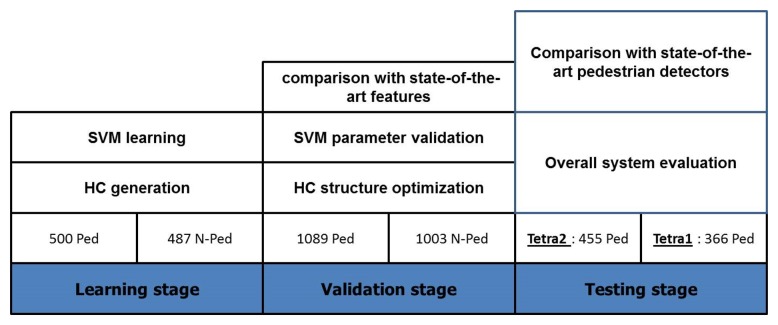
Experimental methodology and evaluation of our system.

From the Tetravision database, we annotated from the FIR images a total of 3900 objects, distributed among learning, validation and testing dataset. The learning images were annotated manually (987 examples) and used for HC generation and SVM learning. For the validation stage, we used a disjoint dataset (2092 examples) for HC structure optimization and SVM parameter validation. In order to perform the overall system evaluation, we used two different image sequences, Tetra1 and Tetra2. Figure 4 describes the evaluation phases. System performances are illustrated by ROC curves (true positive rate over the false positive rate), miss rate vs. false positive rate per image curve and/or F-measures. We analyzed these performance measures using the confidence intervals (CI) at 95%, according to the number of samples in our validation and, respectively, testing sets (see Table 2). A system performs statistically better than another only if the confidence intervals associated with their performance measures are disjoint.

### 7.1. Learning Stage

**Figure 5:**
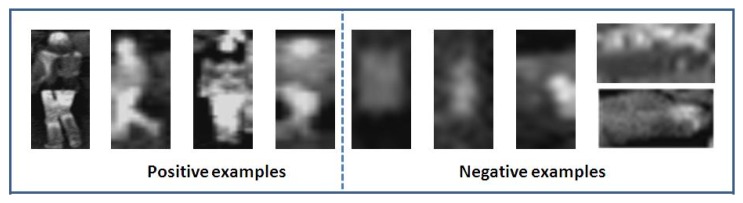
Positive and negative training examples.

First of all, we extracted a subset of representative examples from the learning database to build the HC of pedestrian head appearances. This subset contains only entire (not occluded) pedestrians, which present large variability in appearances, scales and view points. The optimal structure of the HC, corresponding to a tree of five levels, was determined by using our evaluation function (Section 5.1). After HC generation, an SVM classifier was trained to discriminate between Ped and N-Ped on the learning set, which contains 987 examples taken in several situations. Figure 5 shows some examples of images used for SVM learning. One should notice that images presented in this figure are resized just for a standard representation. As we mentioned before, our system performs the classification task without resizing images.

The validation results for HC structure and SVM parameter optimization are presented in the next section.

### 7.2. Validation Stage

The validation stage involves optimizing the HC structure, choosing the appropriate kernel function for SVM and evaluating the discriminative power of our local and global SURF-based features. Moreover, we also compared results obtained with state-of-the-art features within the framework of our pedestrian detection system, where those features were extracted from BB provided by our ROI generation component. All of the results were determined by performing a 10-fold cross-validation scheme in the learning stage to optimize the SVM hyper-parameters before using them on the validation dataset.

#### 7.2.1. HC Structure Optimization

**Figure 6:**
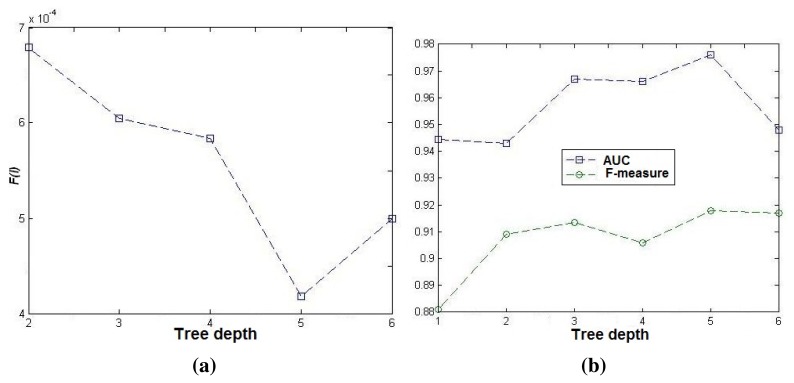
Experimental investigation of the hierarchical codebook (HC) generation. (**a**) *F*(*l*) value *vs.* tree depth; (**b**) classification performance *vs.* tree depth.

Figure 6 highlights that SVM classification performance is largely dependent on the HC structure. In fact, it can be seen in Figure 6b that both the AUC (area under the ROC curve) and F-measure corresponding to the HC structure that we propose outperform those corresponding to a non-hierarchical codebook. In accordance with our evaluation function *F*(*t*_1_, *l*), where *t*_1_ corresponds to the optimized clustering threshold, the estimated structure of our HC corresponds to a tree of five levels (see Figure 6a). The results given in Figure 6b show that our evaluation function allows one to predict the optimal structure of our HC. Moreover, they show that the hierarchical structure allows one to improve the classification results significantly. Note that above five levels, the results are not improved for continuous increasing of the tree depth. This is due to the fact that increasing tree depth could generate irrelevant features, like intermediate clusters similar to negative examples. These features could have less discriminative power, which disturbs the SVM learning.

#### 7.2.2. Kernel and Local/Global SURF Feature Validation

In order to choose the appropriate kernel function for our HC-based local features, we implemented classical (linear and RBF-SVM), histogram-based (HI-SVM and χ^2^-RBF) and local matching (as introduced in [37]) kernels. The results obtained with kernels based on histograms, HI-SVM (AUC = 92.1%) and χ^2^-RBF (AUC = 91.3%) are less satisfactory than those obtained with RBF-SVM (AUC = 92.5%). This kernel function, involving significantly less complexity, performs pedestrian classification much better than LMK. We emphasize the complementarity of local and global SURF-based features, since combining those features yields a significant improvement of 5.1% in the AUC value (from 92.5% to 97.6%). On the validation set, the better performance arises from combining local and global SURF-based features by an RBF-SVM classifier.

#### 7.2.3. Comparison of HC Local/Global SURF Features with the State-of-the-Art in FIR Images

After validating the kernel function for SVM, optimizing the HC structure and evaluating the discriminative power of our HC SURF features (HCS), we compare in Table 3 the classification results obtained with state-of-the-art features. Gabor, Haar, Haar-like and HOG features were extracted from resized BB of 64 × 128 pixels in FIR images. We used four scales and four orientations to compute the mean and the standard deviation of the magnitude of the Gabor transform coefficients. From the Haar wavelet, we obtained a number of 64 features computed at the fourth-level decomposition. While the coefficients of Gabor and Haar wavelets were used with RBF-SVM classifiers, the Haar-like features [17] were trained on a cascade of boosted classifiers with 11 stages. For HOG features, histograms were computed with nine bins on cells of 8 × 8 pixels, having a block size of 2 × 2 cells overlapping by one cell size.

The results presented in Table 3 show that our HC local/global SURF-features achieve, on FIR images, the highest F-measure. The improvement is statistically significant only when compared with Haar and Gabor wavelet-based features, since the corresponding confidence intervals are disjoint.

One should recall that the presented results are obtained within our pedestrian detection framework, since the features were extracted from the BB provided by our ROI generation component. For an overall system comparison, we will consider in the testing stage the state-of-the-art pedestrian detectors, not in the visible spectrum, where they have been proposed, but on FIR images, using Haar-like wavelets with AdaBoost cascade [17], HOG/linSVM [1] and MultiFtr (combination of HOG and Haar) with linear SVM [16].

### 7.3. Testing Stage

In this section, we present experimental results for our overall detection system evaluation. The testing procedure is conducted on two Tetravision image sequences. Statistics reveal a high pedestrian scale variability—Near (80 pixels or more), medium (between 30–80 pixels) and far (30 pixels or less)—And denote an average occlusion rate of 15.5%.

**Figure 7:**
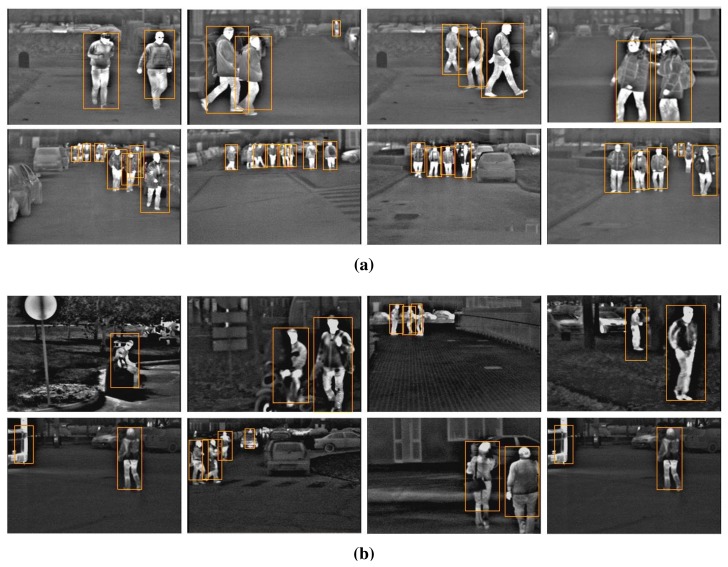
Example of detections from Tetra1 (**a**) and Tetra2 (**b**) image sequences. All images were processed at their original resolution of 320 × 240 pixels.

Figure 7 presents several detection results. It can be seen that most of the pedestrians are correctly detected. Besides, the figure shows that our system is able to detect pedestrians even if they are in the far scale, close to each other or occluded, in the presence of a complex background and in particular situations (sitting, running).

The experimental results, summarizing the performance of our pedestrian detection system in FIR images, are given in Table 4. The results presented are promising and show that our detection system achieves a suitable trade-off between the F-measure and time processing. Even though our current implementation (without coding optimization or parallel computation) does not allow real-time processing, we believe that it is fast, since it process on average more than nine frames per second. The tracking phase improves the pedestrian detection performance in a statistically-significant manner (average of +4.37%).

### 7.4. Benchmark with State-of-the-Art Pedestrian Detectors in FIR Images

We evaluate the performance of our pedestrian detector designed for FIR domain with that of the top performing systems emphasized by recent surveys [10,11] on the Caltech dataset in the visible spectrum. For a fair comparison, we implemented, trained and tested those systems on the same FIR-image datasets and used our pedestrian detector without the tracking stage, since the considered systems do not present such a module. Those state-of-the-art systems are based on the sliding-window approach, and they are: Haar-like wavelets with AdaBoost cascade [17] (VJ), HOG/linSVM [1] (HOG) and MultiFtr within linSVM [16]. Note that a fixed size window is used for the sliding window technique to scan the test images with a scale factor of 1.2 and a step size of 10 pixels. We have found that bootstrapping is essential for obtaining good performance with the HOG and MultiFtr systems. Thus, the classifiers were re-trained on a different and increasingly more difficult set of negative samples, as described in [10].

**Figure 8:**
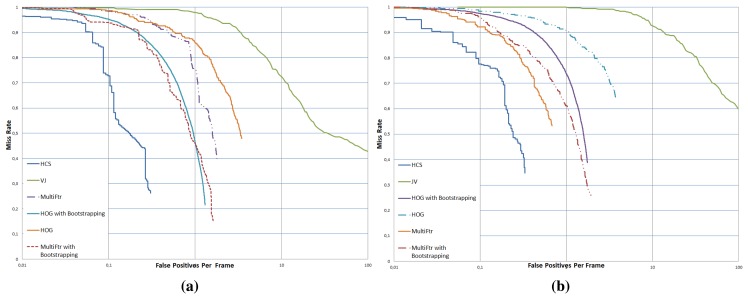
Miss rates *vs.* false positive per-image curves shown for Tetra1 (**a**) and Tetra2 (**b**) from the Tetravision FIR image sequences. Lower curves indicate better performance.

We use the evaluation methodology outlined in [11,12], plotting miss rate vs. false positives per-frame (see Figure 8). The plotting results on our testing dataset show that our detector based on the HC of SURF features (HCS) outperforms the state-of-the-art pedestrian detectors: VJ, HOG and MultiFtr. The benchmark results show that the second best performing detector on our FIR database is MultiFtr with bootstrapping, while the VJ detector shows poor performance. The obtained results demonstrate that sliding-window-based techniques, widely used in the visible spectrum, generate a large number of false positives on FIR data. It is to be noted that the average recall rates obtained by HOG (0.69) and MultiFtr (0.79) with bootstrapping are quite acceptable. On the Tetra1, HOG and MultiFtr with bootstrapping achieves the best recall rate. However, our system achieves the best tradeoff between recall and precision given by an average F-measure of 80.2%. When compared with the second best performing detector (MultiFtr), we achieve a significant performance improvement of 11.4% in FIR images. It is interesting to note that our comparison of state-of-the-art sliding-window pedestrian detectors on the Tetravision FIR dataset are consistent with Dollar *et al.*'s benchmarks [11,12] conducted on the visible Caltech dataset.

### 7.5. Discussion

In the experimental section, we have evaluated the performance of our pedestrian detection system in FIR images by two different sets of experiments. The first set was involved in the validation stage for choosing an appropriate SVM kernel function, optimizing the HC structure and evaluating the discriminative power of our features. Our experiments show that the best performance arises from combining local and global SURF-based features by an RBF-SVM classifier. Additionally, experiments support the exploitation of the hierarchical structure to improve the SVM feature vector and classification results. When compared with state-of-the-art features in FIR images, our SVM-based local/global SURF features provide higher classification performance. This proves that features based on shape or texture extracted by using all pixels within a BB may lose performance, especially if objects are less-textured, not well-centered or occluded.

Our overall detection system was evaluated during the testing stage by a second set of experiments. The results obtained prove the efficiency of our detection system and its potential to handle large-scale and occlusion problems. According to the first set of experiments, the obtained results highlight also the importance of our codebook structure. In fact, the hierarchical structure presents different clustering thresholds, which allows tolerating different degrees of local appearance variability. It is true that this flexibility tends to produce false positives, but SVM enforces stronger constraints and, thus, allows improving the precision of our system. By combining pedestrian detection with tracking in a unified detection system, the robustness was reinforced by increasing the F-measure rate (decreasing the number of false negatives). The system achieves an average F-measure of 84.57% and allows one to process more than nine images per second. Our system is much faster than both HOG/linSVM and MultiFtr, but slower than VJ, which processes 15 images/second. Nevertheless, the results obtained with our system are much better then those provided by the Haar-like Adaboost cascade.

Note that, to the best of our knowledge, no public visible-FIR image dataset acquired with an onboard vehicle camera is available to perform a pedestrian detection benchmark. Recently, [49] proposed a dataset acquired with an Indigo Omega camera, divided into two parts: one that addresses the problem of pedestrian classification and the other the problem of pedestrian detection. Unfortunately, it does not contain any information in the visible spectrum, making a complete assessment of the FIR *vs.* visible domains impossible.

The evaluation of state-of-the-art detectors on the Tetravision dataset reveals that our system is an appropriate solution to the specific problem of detecting pedestrians in FIR images. A limitation of our system is that it fails to detect a pedestrian if his or her head is not visible (*i.e.*, hidden by umbrellas, scarf). This problem is not observed in the dataset. However, we believe that our tracking algorithm can handle the problem of occasional occlusion of pedestrian heads. Another point that we want to mention is that our system is not applicable for high temperature scenes. On a hot summer day, human bodies can appear darker than the background. Even so, our detection system may be applicable by replacing our ROI generation component by a sliding-window algorithm. Further, it will be interesting to investigate more appropriate POI than brightness-based blob detectors.

## 8. Conclusions

In this paper, we discuss and investigate a fast, scale-invariant and robust pedestrian detection system according to the particular conditions of far-infrared daytime images. Our system includes ROI generation, hypothesis classification and pedestrian tracking modules, based all on scale-invariant local feature matching and recognition.

In the first step, pedestrian hypotheses are generated by using a hierarchical codebook, which is a compact representation of the local appearance of pedestrian heads. Then, bounding boxes are constructed and overlapping ones are merged together by using a new formulation of the RNN algorithm. In the second step, we integrate an SVM-based discriminative verification stage that operates on the same codebook representation as the detection stage. Finally, we use a tracking module based on the combination of the mean-shift algorithm with inter-frame SURF features matching. It is to be noted that the SVM classification component attempts to reject the false positive hypothesis from both ROI generation and tracking prediction modules. By combining pedestrian detection and classification with tracking in a unified detection system, our system raises reliability and reduces false alarms significantly.

In order to evaluate our system, we used a traditional training/validation/testing approach. Moreover, we compare our results with the state-of-the-art features and overall pedestrian detectors, which outline the effectiveness of our methodology. The evaluation results show that our detector performs well in detecting pedestrians, even for medium and far scales, but also for partially occluded pedestrians.

The system can still be extended in several ways. For fully-occluded head pedestrian detection, it can be advantageous to generate a second codebook or to include in our HC POI extracted from objects that may cover pedestrian heads. Another possible extensions includes the integration of a visible camera in order to take advantage of the multimodal fusion systems. This solution could be implemented after resolving the task of real-time self-calibration between moving cameras. Robust modality-invariant feature matching for pedestrian detection in visible and FIR images will be a topic of future work.

Alexandrina Rogozan supervised the scientific work presented in this paper, participating actively mostly in the automatic classification task, she proposed the experimental methodology and the statistical analysis of the results.

Adela-Maria Rus participated to the manual annotation of the dataset and the comparison of the proposed detector to the state-of-the-art systems.

Abdelaziz Bensrhair supervised the scientific work presented in this paper and was at the origin of the collaboration with VISLAB laboratory from Italy.

Alberto Broggi was the initiator of this research work, he provided the TetraVision dataset used in the experiments and participated to the optimization of the proposed models.

All the authors participated to the writing of this paper.

## Figures and Tables

**Table 1:** SVM kernels for classifying histogram features. HI, histogram intersection.

**Kernel**	**Definition**
RBF-SVM	$K(X,Y)=\exp(-\frac{1}{2}\frac{{\left\|X-Y\right\|}^{2}}{\sigma^{2}})$
HI-SVM	$K(X,Y)=\sum_{i}\min(|h_{i}{|}^{\beta },|{h^{\prime }}_{i}{|}^{\beta }),\beta \geq 0$
χ^2^	$d_{\chi^{2}}(X,Y)=\frac{1}{2}\times \sum_{i}\frac{{\left(h_{i}-{h^{\prime }}_{i}\right)}^{2}}{\left(h_{i}+{h^{\prime }}_{i}\right)}$
χ^2^-RBF	*K*(*X*, *Y*) = exp(−λ × *d*_χ2_ (*X*, *Y*))

**Table 2:** Performance measures with confidence intervals at 95%.

**Performance m.**	**10%**	**20%**	**30%**	**40%**	**50%**	**60%**	**70%**	**80%**	**85%**	**90%**	**95%**
CI on validation set %	±1.3	±1.7	±2	±2.1	±2.1	±2.1	±2	±1.7	±1.5	±1.3	±0.9
CI on testing set %	±2.1	±2.7	±3.1	±3.4	±3.4	±3.4	±3.1	±2.7	±2.4	±2.1	±1.5

**Table 3:** Comparison with the state-of-the-art features. HOG, histogram of oriented gradients; linSVM, linear SVM.

**Features**	**HC Surf**	**Gabor**	**Haar**	**Haar-Like**	**HOG**
Classifiers	RBF-SVM			boosted cascade	linSVM
F-measure %	**96.2**	87	91	95.43	95.7

**Table 4:** The performance of our overall detection system on Tetravision test sequences.

**Sequence**	**Tracking Phase**	**F-Measure %**	**Time Processing (ms/Frame)**
Tetra1	Included	**88.07**	**92**
	Not included	83.88	91.2
Tetra2	Included	**81.08**	**131**
	Not included	76.53	92.6

## References

[1] Dalal N., Triggs B. Histograms of Oriented Gradients for Human Detection. In.

[2] Jungling K., Arens M. Feature based person detection beyond the visible spectrum.

[3] Bertozzi M., Broggi A., Felisa M., Vezzoni G. Low-level Pedestrian Detection by means of Visible And Far Infra-red Tetra-vision.

[4] Alonso I., Llorca D., Sotelo M., Bergasa L., Del Toro P., Ocana M., Garrido M. (2007). Combination of Feature Extraction Methods for SVM Pedestrian Detection. IEEE Trans. Intell. Transp. Syst..

[5] Gate G., Nashashibi F. Using Targets Appearance to Improve Pedestrian Classification with a Laser Scanner.

[6] Gidel S., Checchin P., Blanc C., Chateau T., Trassoudaine L. Pedestrian Detection Method using a Multilayer Laserscanner: Application in Urban Environment. In.

[7] Fayad F., Cherfaoui V. Tracking objects using a laser scanner in driving situation based on modeling target shape.

[8] Frolov V., Leon F. Pedestrian detection based on maximally stable extremal regions.

[9] Geronimo D., Lopez A., Sappa A., Graf T. (2010). Survey of Pedestrian Detection for Advanced Driver Assistance Systems. IEEE Trans. Pattern Anal. Mach. Intell..

[10] Enzweiler M., Gavrila D.M. (2009). Monocular Pedestrian Detection: Survey and Experiments. IEEE Trans. Pattern Anal. Mach. Intell..

[11] Dollar P., Wojek C., Schiele B., Perona P. Pedestrian detection: A benchmark.

[12] Dollar P., Wojek C., Schiele B., Perona P. (2012). Pedestrian Detection: An Evaluation of the State of the Art. IEEE Trans. Pattern Anal. Mach. Intell..

[13] Dai C., Zheng Y., Li Y. (2007). Pedestrian detection and tracking in infrared imagery using shape and appearance. Comput. Vis. Image Underst..

[14] Andreone L., Bellotti F., de Gloria A., Lauletta R. SVM-based pedestrian recognition on near-infrared images.

[15] Viola P., Jones M., Snow D. Detecting Pedestrians Using Patterns of Motion and Appearance.

[16] Wojek C., Schiele B. A performance evaluation of single and multi-feature people detection.

[17] Viola P., Jones M. (2004). Robust Real-Time Face Detection. Int. J. Comput. Vis..

[18] Sun H., Hua C., Luo Y. A multi-stage classifier based algorithm of pedestrian detection in night with a near infrared camera in a moving car.

[19] Xu F., Liu X., Fujimura K. (2005). Pedestrian detection and tracking with night vision. IEEE Trans. Intell. Transp. Syst..

[20] Suard F., Rakotomamonjy A., Bensrhair A., Broggi A. Pedestrian detection using infrared images and histograms of oriented gradients. In.

[21] Bertozzi M., Broggi A., Gomez C., Fedriga R., Vezzoni G., Del Rose M. Pedestrian Detection in Far Infrared Images based on the use of Probabilistic Templates.

[22] Miron A.D. (2014). Multi-modal, Multi-Domain Pedestrian Detection and Classification: Proposals and Explorations in Visible over StereoVision, FIR and SWIR. Ph.D. Thesis.

[23] Bertozzi M., Broggi A., Carletti M., Fascioli A., Graf T., Grisleri P., Meinecke M. IR Pedestrian Detection for Advanced Driver Assistance Systems.

[24] Binelli E., Broggi A., Fascioli A., Ghidoni S., Grisleri P., Graf T., Meinecke M. A modular tracking system for far infrared pedestrian recognition.

[25] Mahlisch M., Oberlander M., Lohlein O., Gavrila D., Ritter W. A multiple detector approach to low-resolution FIR pedestrian recognition. In.

[26] Meis U., Oberlander M., Ritter W. Reinforcing the reliability of pedestrian detection in far-infrared sensing.

[27] Leibe B., Leonardis A., Schiele B. Combined Object Categorization and Segmentation With An Implicit Shape Model.

[28] Leibe B., Ettlin A., Schiele B. (2008). Learning semantic object parts for object categorization. Image Vis. Comput..

[29] Bertozzi M., Broggi A., Fascioli A., Graf T., Meinecke M. (2004). Pedestrian Detection for Driver Assistance Using Multiresolution Infrared Vision. IEEE Trans. Veh. Technol..

[30] Nanda H., Davis L. Probabilistic template based pedestrian detection in infrared videos.

[31] Apatean A., Rogozan A., Bensrhair A. Objects recognition in visible and infrared images from the road scene.

[32] Sun Z., Bebis G., Miller R. (2006). Monocular Pre-crash Vehicle Detection: Features and Classifiers. IEEE Trans. Image Process..

[33] Papageorgiou C., Evgeniou T., Poggio T. A Trainable Pedestrian Detection System.

[34] Lowe D. (2004). Distinctive Image Features from Scale-Invariant Keypoints. Int. J. Comput. Vis..

[35] Bay H., Tuytelaars T., Gool L. Surf: Speeded up robust features.

[36] Wallraven C., Caputo B., Graf A. Recognition with Local Features: The Kernel Recipe.

[37] Fritz M., Leibe B., Caputo B., Schiele B. Integrating representative and discriminant models for object category detection.

[38] Lampert C.H., Blaschko M.B., Hofmann T. Beyond sliding windows: Object localization by efficient subwindow search.

[39] Belongie S., Fowlkes C., Chung F., Malik J. Spectral Partitioning with Indefinite Kernels Using the Nyström Extension.

[40] Boughorbel S., Tarel J.P., Boujemaa N. (2005). Generalized Histogram Intersection Kernel for Image Recognition. Proc. IEEE Int. Conf. Image Process..

[41] Besbes B., Labbe B., Rogozan A., Bensrhair A. SVM-based fast pedestrian recognition using a hierarchical codebook of local features.

[42] Grauman K., Darrell T. (2007). The Pyramid Match Kernel: Efficient Learning with Sets of Features. J. Mach. Learn. Res..

[43] Mikolajczyk K., Schmid C. (2004). Scale and Affine Invariant Interest Point Detectors. Int. J. Comput. Vis..

[44] Rosenberg A., Hirschberg J. V-Measure: A Conditional Entropy-Based External Cluster Evaluation Measure.

[45] Mikolajczyk K., Leibe B., Schiele B. (2006). Multiple Object Class Detection with a Generative Model. IEEE Conf. Comput. Vis. Pattern Recogn..

[46] Mikolajczyk K., Schmid C., Zisserman A. Human detection based on a probabilistic assembly of robust part detectors.

[47] Comaniciu C., Meer P. (2002). Mean shift: A robust approach toward feature space analysis. IEEE Trans. Pattern Anal. Mach. Intell..

[48] Witten I., Frank E. (2005). Data Mining: Practical Machine Learning Tools and Techniques.

[49] Olmeda D., Premebida C., Nunes U., Armingol J., de la Escalera A. (2013). Pedestrian detection in far infrared images. Integr. Comput. Aided Eng..

